# Method Development and Validation of an Aerosol Sampling Technique for the Analysis of Nicotine in Electronic Cigarette Aerosols

**DOI:** 10.3390/molecules29153487

**Published:** 2024-07-25

**Authors:** Maarten Dill, Eric Deconinck, Sophia Barhdadi

**Affiliations:** Sciensano, Scientific Direction Chemical and Physical Health Risks, Service of Medicines and Health Products, J. Wytsmanstraat 14, B-1050 Brussels, Belgium; maarten.dill@sciensano.be (M.D.); eric.deconinck@sciensano.be (E.D.)

**Keywords:** nicotine, e-cigarette, aerosol, method validation

## Abstract

Because of the increasing popularity of e-cigarettes, monitoring the e-cigarette market has become important for national health authorities to guarantee safety and quality. In the EU, the Tobacco Products Directive requires emission studies for e-cigarette products. The absence of industry guidelines for studying these emissions and the lack of proper validation in the literature led us to develop and validate a method using the total error approach for the determination of nicotine in e-cigarette aerosols. A commercial vaping device was used to generate aerosols, which were then collected on Cambridge filter pads and measured for nicotine concentration by UHPLC-DAD after extraction. The method was successfully validated by generating accuracy profiles, which show that the β-expectation tolerance intervals remained below the acceptance limits of ±20%. Within-run repeatability and intermediate precision were considered acceptable since the highest RSD value obtained was below 5%. The method was applied to 15 commercial e-liquids. A complete validation of a method for the analysis of e-cigarette emissions is presented, including several parameters that impact the accuracy and reproducibility. Similar systematic approaches for method development and validation could be used for other e-cigarette emission analysis methods to ensure the reliability of the measurements.

## 1. Introduction

E-cigarettes have been marketed as a less harmful alternative to tobacco cigarettes and as a potential smoking cessation aid. They entered the global market in 2006, three years after Hon Lik invented the modern version of the e-cigarette [1]. Their popularity is shown by Statistica, which states that the global revenue of e-cigarettes rose from USD 16.0 billion to USD 24.6 billion from 2018 to 2023 [2]. Within the EU, the latest Eurobarometer survey published in 2021 reports that 2% of EU respondents currently use e-cigarettes [3]. Nevertheless, the long-term toxicological health effects of e-cigarettes remain unclear, and researchers’ opinions appear to be mixed but heavily influenced by relationships with the industry [4].

An e-cigarette aerosolizes a mixture of propylene glycol (PG), glycerin (G), and sometimes water or ethanol. Nicotine and flavorings are often added to this mixture to modulate the user experience [5,6]. The operational procedure involves passing an electric current through a coil of specific resistance, generating heat in the process. The coil reaches hundreds of degrees Celsius [7], causing the e-liquid to aerosolize and allowing the generated aerosol to be inhaled.

Various international regulatory policies are established to control the sales, ingredients, and marketing restrictions of e-cigarette products. Implementing the regulations for e-cigarettes is challenging in certain aspects because of the wide variety of available e-cigarette devices and e-liquid characteristics [8]. The quality assurance of e-cigarettes includes monitoring emission requirements, which depend on the ingredients of the e-liquid and the performance of the e-cigarette device. Emission studies are required by the EU Tobacco Products Directive for all Member States of the EU [9]. Manufacturers and importers of e-cigarette products must notify and submit key information on their products to the competent authorities of the Member States where they intend to market them. In Article 20 §2b, the TPD states that this information also includes “a list of all ingredients contained in, and emissions resulting from the use of the product, by brand name and type, including quantities thereof”. This includes all components found in the emissions, or at least the most critical ones such as nicotine, toxic carbonyl compounds, and metals. Other emission-related information that the manufacturer must provide and that is required to appear on the unit packages and any outside packaging of electronic cigarettes is “an indication of the nicotine content of the product and the delivery per dose” (Article 20 §4bi). The Member States’ health authorities, in turn, must ensure and monitor that “electronic cigarettes deliver the nicotine doses at consistent levels under normal conditions of use” (Article 20 §3f) [9]. Similar emission requirements can be found in the U.S. [10,11].

The European standardization bodies published a document (CEN/TR 17236:2018) specifying the constituents that should be measured in the aerosol of vaping products [12]. Although no analytical reference method is given, it is noted that a standardized vaping regime should be used to allow comparison among different products. Nicotine is also expected to be measured in the emissions to demonstrate the consistency of delivery and a potential measure for normalization. In the case of prefilled e-liquids, the entire product can be analyzed as such. However, when evaluating e-liquids sold in refill cartridges or when assessing the device itself, it is essential to establish a reference device and reference e-liquid. This CEN/TR 17236:2018 document also refers to national standards such as the French standard XP D 90-300 [13]. In this document, limit values are also given for each parameter besides the substances to be measured in the emissions. In the case of nicotine emissions, the nicotine concentration measurement for each of the series must be within a range of ±30% of the mean value of the series. There are also guidelines and standards that have been established by or in collaboration with the industry. The ISO 20768:2018 guidelines are based on CORESTA’s recommended method n° 81 (CRM81) and propose instructions for generating these aerosols [14,15]. While many research groups use these puffing parameters, some argue that they are more representative of cigarette users and lower-power devices, not systems designed for more intense vaping behaviors [16]. On the other hand, they may still be useful for the quality testing of devices and e-liquids. ISO/DIS 24199 also covers the determination of nicotine in vape product emissions and suggests using glass fiber filter pads as a collection medium for the aerosol [17]. Different methodologies have been described for the analysis of nicotine in e-cigarette emissions for various purposes, for example, method development [18,19,20], market studies [21,22,23], and in vitro [24,25,26,27] and in vivo studies [28]. Other researchers made efforts to investigate factors that influence emissions characteristics including nicotine transfer [29,30,31]. Typically, e-cigarette emission studies focus on the validation of the quantitative analysis step, while the validation of the aerosol sample preparation method is often overlooked.

The purpose of this study was to develop and validate a straightforward and robust sample preparation method for the quantification of nicotine in aerosols in order to facilitate the quality control of nicotine concentrations in aerosols. Several steps in the process of e-cigarette aerosol sample preparation were investigated, as well as the different parameters that might influence these. To further prove the applicability of the method, the aerosols of commercial e-liquid samples were analyzed, and the consistency in nicotine delivery was investigated.

## 2. Results and Discussion

### 2.1. Method Development and Optimization

In contrast to other research groups, who built and validated their own unique smoking machine to generate e-liquid aerosols, it was decided to use a commercially available device [32,33,34]. Before generating aerosols, the volume of each puff was calibrated using a soap bubble meter. Calibration was also performed between puff sessions, if necessary. It was noticed that leaving the vaping machine on contributed to keeping a consistent puff volume. In instances where the machine was not left in an activated state, a gradual decline in puff volume was observed, resulting in the need for recalibration following the initial puffing events upon activating the smoking machine despite respecting the recommended 30 min warm-up period. Aerosols were generated utilizing the puff topography described in the CRM81, which recommends a puff duration of 3 s, a puff volume of 55 mL, and an inter-puff duration of 30 s [15]. Although CORESTA involves collaboration among different stakeholders in the tobacco industry [35], and the proposed puffing parameters may not be appropriate for all e-cigarette devices, its guidelines are useful for comparative nicotine dosimetry studies involving different devices and e-liquids since they have been extensively used by researchers in the field. The decision was made not to pre-heat the heating element prior to the aerosolization tests because of the observation of a constant aerosol production over time, which indicated no gradual temperature increase.

**Figure 1:**
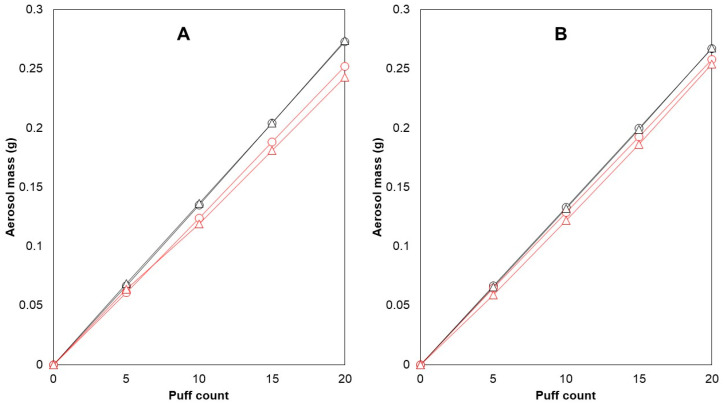
CFP saturation testing and within-brand differences in the reference device and coil. E-cigarette 1 and e-cigarette 2 were used to vaporize e-liquid C with 1.0 ohm coils. Aerosol generation occurred in a linear fashion during collection on the filters (R^2^ ≥ 0.9995). After 20 puffs, e-cigarette 1 consistently produced 8.247% more aerosol than e-cigarette 2 (**A**). The difference reduced to 3.605% after switching coils (**B**). Legend: mass generated (g)—e-cigarette 1 (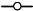), mass generated (g)—e-cigarette 2 (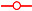), mass collected (g)—e-cigarette 1 (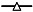), mass collected (g)—e-cigarette 2 (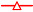).

Cambridge filter pads (CFPs) were used to collect the generated aerosols and were able to collect almost all of the generated aerosol mass. The recoveries of the trapped aerosol on the CFP were calculated as the percentage of the weight of aerosol trapped on the filter versus the weight difference in the e-cigarette before and after puffing. These values were between 98.9 and 101.4% (*n* = 12), which also correspond to previous findings [36]. In a preliminary test with 10 puffs per puffing session (*n* = 23), an average aerosol mass of 125.5 ± 6.3 mg was collected on the filter. Later, the choice was made to double the puff number, resulting in better repeatability of collected aerosol mass (5.0% mean RSD for 10 puffs, 3.5% mean RSD for 20 puffs). The possibility of CFP saturation after 20 puffs was investigated as well (Figure 1).

After 20 puffs, the linear relationship between the number of puffs and aerosol mass trapped on the CFPs indicated that there was no saturation of the CFP. An alternative option for aerosol collection would be to use impingers. It has been demonstrated that both filter pads and impingers are able to collect nicotine; however, one research group noticed that the absolute intensity of the nicotine response from a filter pad was 15% higher than that from an impinger [37]. Moreover, it was previously determined that the use of three impingers in series was necessary to collect all of the aerosolized nicotine [19]. Thus, impingers were found to be more time-consuming and less convenient to use compared with filter pads when considering the rinsing required to ensure maximum aerosol recovery, as it is possible for a significant portion of the aerosol to condense on the inner glass wall. Therefore, CFPs were used instead of impingers for the collection of the aerosols.

**Figure 2:**
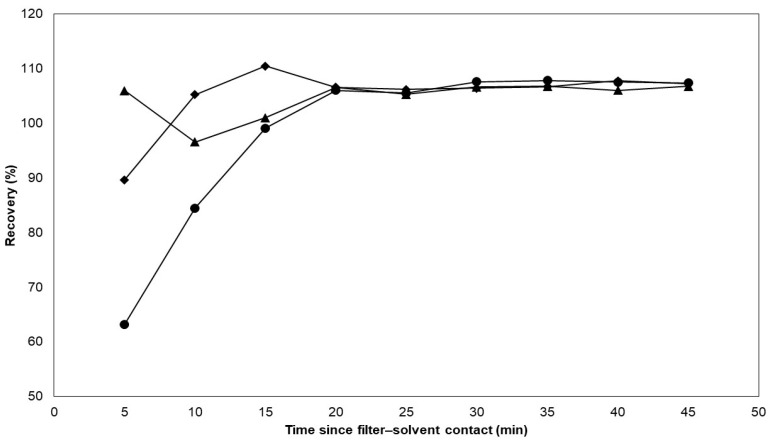
The extraction capacity of different solvents on CFP-spiked e-liquid was evaluated. The spiked filters were immersed in three different solvents including methanol, acetonitrile, and ammonium borate buffer. Each data point represents a single sampling event. Methanol and acetonitrile exhibited higher extraction rates compared with the ammonium borate buffer. After 20 min, all three solvents showed similar extraction capacities. Legend: methanol (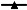), acetonitrile (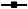), ammonium borate buffer (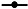).

Next, the appropriate extraction solvent was selected for the extraction of nicotine from the filter pads, and, in the same experiment, the extraction rate was determined for the different solvents. The CFPs were spiked with approximately 0.160 g of e-liquid C. The filter pads were then immersed in separate Erlenmeyer flasks each containing 20.0 mL of methanol, acetonitrile, and ammonium borate buffer. Every five minutes, from the start of the experiment until 45 min, 0.5 mL of the extraction mixture was taken and subsequently diluted to 5.0 mL using Milli-Q water to determine the nicotine concentrations. The extraction recoveries are plotted against the extraction time in Figure 2.

The results show that the extraction rate appears to dominate for methanol and acetonitrile over the ammonium borate buffer. However, the results also suggest that all three solvents have the same intrinsic extraction capacity after 20 min, which is consistent with the extraction duration utilized in a past study for nicotine extraction from e-cigarette aerosols [18]. In order to achieve the best possible yields within an appropriate time range, the duration of extraction was extended to 30 min while shaking at 200 rpm. Ultimately, the ammonium borate buffer was deemed the optimal choice because of its compatibility with the mobile phase. After extracting, the solution was filtered through a mixed cellulose filter with a 0.45 µm pore diameter. For other applications, the use of saline [20] and buffers such as phosphate buffers [18] are also popular choices for the extraction of nicotine from CFPs because of their compatibility with cell cultures [38]. Furthermore, organic solvents such as methanol [23], isopropanol [39], and ethyl acetate [40] have also been used to extract nicotine from CFPs because they are more compatible with GC-MS and GC-FID analyses.

Carry-over from the e-liquid tank of the device was investigated. A protocol was developed for cleaning the tanks (exclusive of the atomizer), which included a thorough rinse with detergent and Milli-Q water (Millipore, Billerica, MA, USA), followed by the application of denatured ethanol (96%, VWR, Rosny-sous-Bois, France) and drying. The efficacy of the protocol was verified by first vaping (10 puffs/session) three randomly selected commercial e-liquids, followed by performing the cleaning protocol after each vaping session, then vaping e-liquid C with fresh coils, followed by extraction of the CFP. Upon analysis of the three extracts, no quantifiable nicotine peak or other contaminants were identified using Ultra-High Performance Liquid Chromatography with a Diode-Array Detector (UHPLC-DAD) (Waters, Milford, CT, USA).

### 2.2. Method Validation

#### 2.2.1. Accuracy Profiles

**Figure 3:**
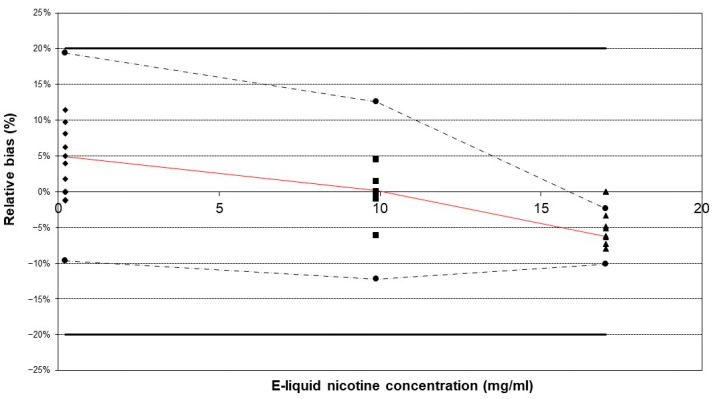
Accuracy profiles of the method validation outcomes. The three reference e-liquids were vaporized using the developed method in triplicate and repeated over three separate days. The tolerance intervals for β-expectation did not exceed the acceptance limits of ±20%. Legend: relative bias (–), upper and lower β-expectation limits (–·–·) for each concentration (●), upper and lower acceptance limits set at 20% (–), relative back-calculated concentrations for e-liquid A (♦), e-liquid B (▲), and e-liquid C (■).

Using a qualified commercial vaping device does not imply that aerosol generation is validated, as it also depends on the characteristics of the e-cigarette device itself. By validating the sample preparation method, the aerosol generation is indirectly validated. Method validation was subsequently performed in accordance with the International Council for Harmonisation of Technical Requirements for Pharmaceuticals for Human Use (ICH) guidelines [41]. To validate the method, the main attributes that needed to be validated were accuracy and precision, as was performed during the validation of the chromatographic method for the analysis of e-liquids. This means that selectivity, LOD, LOQ, linearity, and the matrix effect were not re-validated since these validation parameters are related to the detection method. Despite the likelihood of alterations in the matrix composition of the e-liquid following aerosolization, the analytical method has been validated for a broad range of PG/G ratios, which represent the primary matrix component [42]. The accuracy and precision of the methodology for e-cigarette aerosol were successfully validated using the “total error” approach. The obtained accuracy profiles indicate that the tolerance intervals for β-expectation did not exceed the acceptance limits of ±20% (Figure 3).

This result implies that future measurements will have a 95% probability of falling within the bias limits of [−20%, 20%]. The highest mean relative bias (−6.26%) was observed at the highest concentration level. The within-run repeatability and intermediate precision were acceptable, with the highest RSD being less than 5% (Table 1).

#### 2.2.2. Robustness of the Method

**Figure 4:**
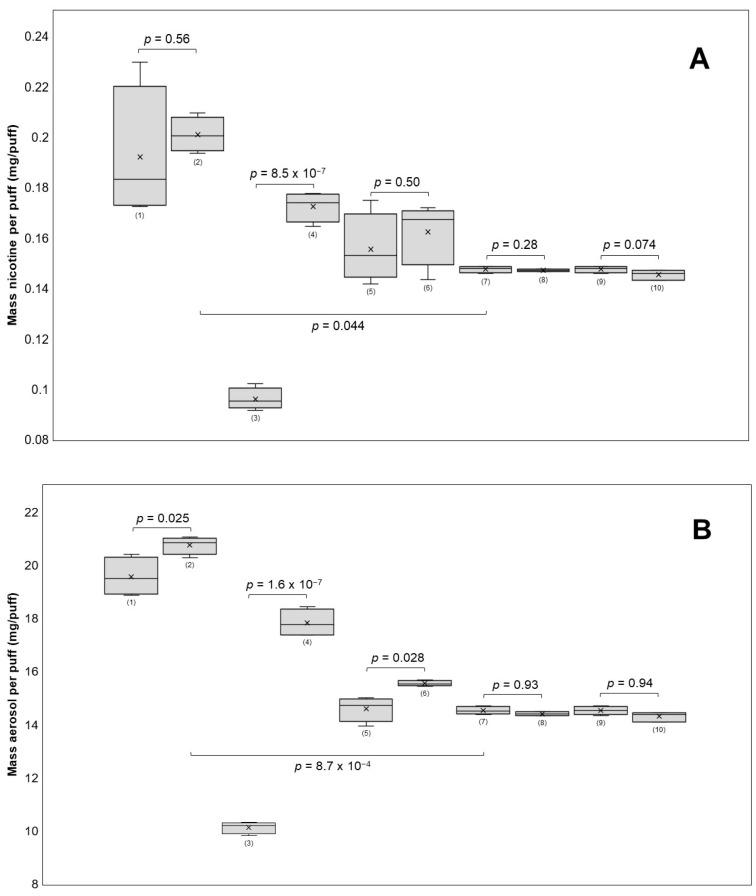
The influence of different puff parameters on nicotine recovery (**A**) and aerosol recovery (**B**) was tested. The puffing parameters used for each group were (power (W)/puff volume (mL)/puff duration (s)/IE = immediate extraction or 24E = extraction after 24 h/FF = front side filter or BF = back side filter): (1) 15/55/3/IE/FF, (2) 20/55/3/IE/FF, (3) 10/55/2/IE/FF, (4) 10/55/4/IE/FF, (5) 10/50/3/IE/FF, (6) 10/60/3/IE/FF, (7) 10/55/3/IE/FF, (8) 10/55/3/24E/FF, (9) 10/55/3/IE/FF, and (10) 10/55/3/IE/BF. A 30 s inter-puff duration was used in all groups. The nicotine yields per puff were significant when the puff duration was changed and when the groups using 10 W were compared with those using 15 W (**A**). Aerosol yields per puff changed significantly with puff duration, power, and puff volume (**B**).

The robustness of the method was evaluated by varying the puff volume (±5 mL), puff duration (±1 s), and e-cigarette power settings (±5 W) and filter position. If the respective parameter underwent any variation, the remaining parameters of the validated method were unchanged (e.g., when changing the puff volume, the puff duration and power settings were not altered). The results shown in Figure 4 indicate that the nicotine yields significantly increased from 0.0960 to 0.1725 mg nicotine per puff (*p* = 8.5 × 10^−7^) when increasing the puff duration from two to four seconds.

The puff volume did not significantly affect (*p* = 0.50) nicotine transfer (50 mL: 0.1556 mg nicotine/puff; 60 mL: 0.1624 mg nicotine/puff), but it significantly (*p* = 0.028) impacted aerosol mass transfer (50 mL: 14.60 mg aerosol/puff; 60 mL: 15.55 mg aerosol/puff). Furthermore, the aerosol mass per puff increased significantly (*p* = 1.6 × 10^−7^) from 10.13 to 17.83 mg per puff. Although the power increased from 15 W to 20 W, the nicotine yields did not change significantly (*p* = 0.56) (0.1921 to 0.2010 mg nicotine/puff on average). However, there was a significant increase (*p* = 0.025) in the transfer of aerosol mass, averaging an increase of 19.57 to 20.76 mg aerosol/puff. In comparison, all four groups using the puff topography of the validated method, which includes 10 W, had significantly lower nicotine levels per puff than the 15 W and 20 W groups. Nicotine concentrations were not significantly affected by any of the investigated parameters, likely because of a correlation between nicotine and aerosol mass transfer. It is generally found that puff duration [29,40,43] and power [40,43,44,45] are major factors influencing both aerosol and nicotine yields. Both factors raise the coil temperature, which is directly related to the amount of aerosol produced [46]. In contrast, the puff volume does not seem to have a clear impact on either outcome variable [39,43]. One research group found that an increase in puff volume from 20 mL to 150 mL affected nicotine delivery but not aerosol transfer in the two e-cigarette devices they tested, suggesting that large changes in puff volume may have an effect on nicotine yields [29]. It is important to note that modifying the puffing parameters led to a decrease in the overall repeatability of the method. The nicotine yields per puff for all four groups using the validated puffing regime had RSDs of less than 1.6%. However, the RSDs for the groups using a modified puffing regime ranged between 3.4% and 14%. This implies that the method should be re-validated when drastically changing the puff parameters, which is an important limitation of this method.

It was found that changing the position of the CFPs (front and back) did not have a significant effect on nicotine content per puff or aerosol mass transfer. Two e-cigarette devices were tested for their ability to produce consistent amounts of aerosol. E-cigarette 1 generated more aerosol with each puff, resulting in 8.2% more aerosol after 20 puffs compared with e-cigarette 2 (Figure 1A). To investigate whether the coil was responsible for this difference, both coils were switched between the devices, and the experiment was repeated. The difference declined to 3.6%, suggesting that there was variability in performance between the same models of devices and coils (Figure 1B). These variations in aerosol generation performance within the same brand have been previously reported in the literature [47] and underscore the importance of reference devices when evaluating e-liquids and the need to test multiple units when assessing e-cigarette devices of the same brand.

Next, the stability of the collected analyte on the CFP and the vaped aerosol extract was evaluated. It was noticed that whether the CFP was immediately extracted or after 24 h, it did not cause any significant difference in the final nicotine yield. Others have reported longer storage periods in which nicotine-containing aerosols collected on CFPs were effectively stored at −80 °C for 30 days [48]. To test the stability of nicotine in the ammonium borate buffer, three aerosol extracts generated during the method application were stored appropriately and analyzed to investigate the influence of storage time and temperature on the estimated nicotine dose per puff. The study found that nicotine remains stable in the ammonium borate buffer for up to seven days, regardless of the storage conditions.

### 2.3. Method Application

**Figure 5:**
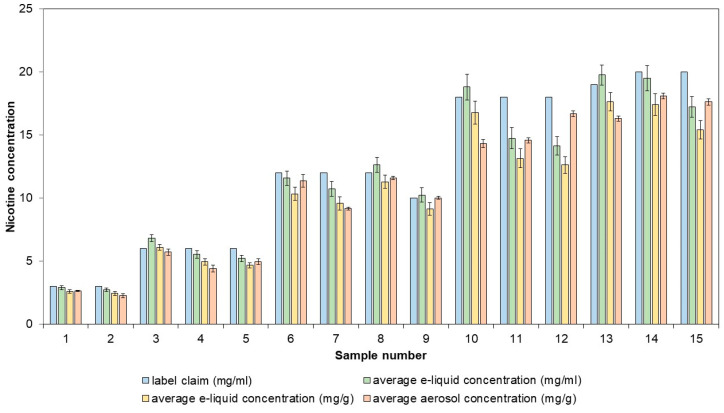
Labeled nicotine concentrations compared to measured nicotine concentrations in commercial e-liquids and their corresponding aerosols. The aerosols were generated, collected, and extracted using the validated sample preparation method. UHPLC-DAD was utilized to quantify the nicotine concentrations.

The applicability of the method was demonstrated with the analysis of 15 e-liquids, which were vaped using the validated method. Both the nicotine concentration in the e-liquid and in the aerosol were determined (Figure 5).

The e-liquids and their respective aerosols had similar nicotine concentrations, with recoveries ranging from 92.79% to 109.72%. Of the 15 e-liquids tested, five had nicotine concentrations that were more than 10% lower than the labeled values. Direct determination of nicotine concentration in e-liquids is sufficient for label verification and does not need to involve aerosol generation. The determination of the nicotine concentration in aerosols is important when assessing consistency in nicotine delivery. For three of the previously investigated samples, five puffing blocks of 20 puffs were taken, and for each puffing block, the nicotine concentration was determined (Table 2). The highest nicotine concentration deviation from the average was −8.11%, which still complies with the ±30% limit values.

It is important to emphasize that when considering nicotine exposure in humans, aerosol nicotine concentration is a poor indicator because it ignores the total aerosol mass transfer. Also, when assessing nicotine yield, the results from such studies should be treated with caution since these values are highly dependent on puff parameters and may not represent realistic values for each individual. To ensure objectivity, correction factors based on realistic dose shifts observed in various nicotine dosimetry studies that measure the effects of power and puff duration could be introduced. These correction factors can be used to generate minimum and maximum values that would likely encompass the possible nicotine exposure to an individual using the corresponding e-liquid. A similar concept was previously proposed, which involved the use of nicotine flux (mass of nicotine per second) as a regulatory tool instead of mass of nicotine per puff [49]. Furthermore, our method could also be used in the context of quality control studies to compare the performance of different devices using reference e-liquids, and vice versa. Finally, the method could also be used as a foundation for studying the transfer characteristics of aerosols and nicotine, as well as any potential influences of unknown factors on these outcome variables.

## 3. Materials and Methods

### 3.1. Standards and Reagents

The reference standard (-)-nicotine (≥99%) was purchased from Merck (Darmstadt, Germany). The matrix components propylene glycol (PG) and glycerol (G) were provided by Fagron (Nazareth, Belgium). Methanol (HPLC grade) was obtained from Biosolve (Valkenswaard, the Netherlands), and concentrated ammonia (28%) was purchased from VWR (Rosny-sous-Bois, France). Boric acid (≥99.5%) was purchased from Sigma (Schnelldorf, Germany). Milli-Q water was prepared using a Milli-Q Gradient system (Millipore, Billerica, MA, USA).

### 3.2. Standard Solution Preparation

A nicotine stock solution of 1.000 mg/mL was prepared in methanol and then further diluted with Milli-Q water to create a calibration line comprising four concentration points in the range of 0.002 to 0.02 mg/mL. A control solution of 0.01 mg/mL was prepared using the same procedure. These solutions were prepared in amber volumetric flasks and stored at 4 °C for up to one week.

### 3.3. Preparation of Spiked Matrix Validation Samples

For the method validation, spiked e-liquids were prepared containing PG, G, and nicotine in three different concentrations. Previously, it was found that e-liquids that were labeled to contain no nicotine actually had nicotine concentrations of 0.2 ± 0.05 mg/mL [50]. It was decided to prepare an e-liquid with this concentration to test the capacity of this method to quantify nicotine in e-liquids with small traces of nicotine (e-liquid A). In addition, an e-liquid was produced that approached a high nicotine concentration of 18 ± 2 mg/mL (e-liquid B), following the maximum allowed concentration of 20 mg/mL established by the EU [9]. A third e-liquid was made with a medium nicotine concentration of 10 ± 2 mg/mL (e-liquid C). The e-liquids were prepared by weighing an appropriate amount of nicotine and diluting it with a homogeneous mixture of 50% m/m PG/G. After shaking the e-liquid thoroughly, the mixture was sonicated for 15 min to remove air. The e-liquids were then placed in the freezer for storage.

### 3.4. Aerosol Sample Generation and Collection

Aerosols were generated using a CETI-8 smoking machine (Cerulean, Milton Keynes, UK) equipped with button activation switches. It was not possible to introduce a tilted angle, as this option was not available to us. The e-liquid samples were aerosolized using an Aspire Zelos X device (Shenzhen, China) with a Nautilus 3 clearomizer at a power of 10 W with a 1.0 Ohm mesh coil. The largest opening in the airflow control ring was selected. For each e-liquid, a new coil was used to avoid contamination. Aerosols were collected on 44 mm CFP (Körber, Hamburg, Germany). Before each vaping session, it was ensured that the tank was fully filled with e-liquid and that the battery was fully charged. A 15 min period was introduced to allow the saturation of the wick. For each sample, 20 puffs with a puff volume of 55 mL and puff duration of 3 s were taken with a 30 s break between each puff. A square wave puffing profile was selected. Prior to vaping a new set of samples, the puff volume was calibrated using a soap bubble meter (Cerulean, UK). A five-minute rest period was included after puffing to ensure complete condensation of the aerosol on the CFP. The filter and holder were weighed before and after puffing to determine the mass of the collected aerosol. The filters were then immersed in 20.0 mL of 0.01 M ammonium borate buffer solution (pH = 9.0) and shaken at 200 rpm for 30 min using a universal shaker (Edmund Bühler, Bodelshausen, Germany). The extracts were filtered through 25 mm mixed cellulose ester syringe filters with a 0.45 µm pore diameter (BGB^®^, Böckten, Switzerland). Extracts were stored at 4 °C for a maximum of seven days. For the method validation, the samples were analyzed on the day of sample preparation. For extracts generated from e-liquids above 1 mg/mL, an additional dilution step was required to fit in the quantifying range.

### 3.5. Chromatographic Analysis

The nicotine levels in the aerosol extracts were quantified in accordance with a previously validated method [42]. In summary, an Acquity UPLC^TM^ system was utilized, equipped with a photodiode array detector, an Acquity BEH RP C18 2.1 mm × 100 mm, 1.7 µm column, and a Van Guard BEH RP C18 1.7 µm pre-column (Waters, Milford, CT, USA). The mobile phase was composed of 0.01 M ammonium borate buffer (pH = 9.0) and acetonitrile, which was gradient eluted at a flow rate of 0.4 mL/min and a column temperature of 30 °C. The buffer–acetonitrile ratio was 95:5 for the initial minute, 75:25 between minutes seven and nine, and 90:10 for minutes nine and a half to eleven. Throughout the analysis, the sample temperature was maintained at 10 °C. The injection volume was set at 10 µL in full loop mode. Each sample was injected twice to account for potential system bias. Additionally, the four calibration solutions and the control solution were injected twice as well, both before and after each sample run.

### 3.6. Method Validation

Validation of the aerosolization, trapping, and extraction of the methodology was conducted using the “total error” approach in accordance with the validation requirements of ISO-17025 to confirm the fitness-for-purpose of the method [51]. The total error approach is based on accuracy profiles and ensures the suitability of a method by demonstrating that a percentage of future results (β-expectation tolerance) will fall within predefined acceptance limits. In order to estimate this expected proportion of future observations, the total error of an analytical method has to be taken into account. The total error is the difference between the observed value and the true value and is due to both the systematic error and the random error. For the method validation, the β-expectation tolerance limits were calculated at 95%. At the moment, there is no agreement on the acceptance limits that should be used for e-cigarette analysis. An acceptance limit of 10% has been used for the determination of nicotine in e-liquids. Since 10% was accepted for e-liquids and the different guidelines accept 30% variation [13], an acceptance limit of 20% was chosen for aerosol sampling.

The three validation samples (e-liquid A, e-liquid B, and e-liquid C) were aerosolized in triplicate and analyzed with UHPLC-DAD for 3 consecutive days. The accuracy and precision were determined for each concentration level. Accuracy profiles were built by plotting the concentration levels against the recoveries (%), with the acceptance limits and the upper and lower tolerance limits. Recoveries were calculated by comparing the aerosol concentrations to the corresponding e-liquid concentrations.

The robustness of the method was investigated by evaluating several factors that may influence the nicotine dosage per puff, the mass of generated aerosol, and the nicotine concentration in the aerosols. The puff volume was varied ±5.0 mL from the validated value and the puff duration was varied ±1.0 s. Different power settings were tested for the e-cigarette device (15 W, 20 W). Besides the parameters that influence the production of the aerosol, the filter position in the holder was also tested since the filters used for capturing the aerosol had a smooth and rough side. Additionally, the influence of the device was assessed by comparing the aerosol generation of two e-cigarette devices and two 1.0 ohm coils. Parameters were assessed individually in quadruplicate, and each factor’s significant value (*p* < 0.05) was calculated using F-tests followed by the appropriate t-tests. The other validation parameters such as selectivity, the matrix effect, linearity, the limit of detection (LOD), and the limit of quantification (LOQ), which are associated with the chromatographic method, have previously been validated and are similar for the determination in the aerosol matrix [42].

The stability of the collected analyte on the CFP and the vaped aerosol extract was assessed. For the first test in this stability study, the aerosol collected in the filter was immediately extracted and analyzed using the chromatographic method on the same day. The remaining filter pads were stored in the extraction flask for 24 h before extraction. In the second test, analyte stability in the extractions was evaluated by re-analyzing aerosol extracts from three different samples prepared for the application of the method after seven days (room temperature, refrigerator, and freezer).

### 3.7. Method Application

E-liquid samples ranging from 3 mg/mL to 20 mg/mL were obtained from inspections conducted by the federal government authorities at various vaping shops in Belgium. All samples were stored at room temperature and protected from light. Each individual e-liquid was puffed in quadruplicate with the same coil (1.0 ohm). Aerosol extracts of each e-liquid sample were analyzed in duplicate. The nicotine concentrations of the e-liquid samples were also analyzed using the same chromatographic method to calculate the recoveries. Additionally, three commercial e-liquids were randomly selected to test the consistency of nicotine delivery. For this, the nicotine concentration (mg/mL) was determined for a consecutive of 5 series of 20 puffs.

## 4. Conclusions

The aim of this study was to develop and validate a straightforward sample preparation method for the analysis of e-liquid aerosols. Aerosols were generated using a commercial smoking machine, collected on CFPs, extracted in an ammonium borate buffer, and analyzed by UHPLC-DAD. The method was successfully validated using accuracy profiles. The validation results indicated that the tolerance intervals for β-expectation did not exceed the acceptance limits of ±20%. The robustness tests demonstrated a significant increase in nicotine yields per puff when the puff duration was extended from 2 to 4 s, as well as when comparing groups using 10 W with those using 15 W. Aerosol yields per puff were also found to significantly change as a function of puff duration, power, and puff volume. It is important to note that the method has the limitation of requiring re-validation if there is a deviation from the validated puffing parameters. The method was applied to 15 commercial e-liquids to measure the nicotine concentration in collected aerosols. Nicotine consistency testing was also performed where a maximum deviation of 8.11% from the mean nicotine concentration in the emission was found. These tests are important for compliance with the Tobacco Products Directive (2014/40/EU), which requires nicotine dosages to be reported and also to be consistent. To conclude, this method can be used not only in nicotine dosimetry studies and market research but also as a basis for investigating aerosol transfer phenomena and any potential influences of unknown factors on nicotine and aerosol exposure. The need to obtain quantitative data on e-liquid aerosols comes along with the responsibility to validate the entire method, including aerosolization, capturing, and extraction procedures. This validation method can be used as a basis for such procedures, thus encouraging other researchers to validate their methods. This would be an important step towards the standardization of methods for reporting compound doses in e-liquid aerosols.

## Figures and Tables

**Table 1:** Corresponding data of the accuracy profiles.

	Accuracy (Total Overall Bias %)			Repeatability (RSD)			Intermediate Precision (RSD)			β-Expectation Tolerance Limit		
Concentration (mg/mL)	0.2 ± 0.04	10 ± 2	20 ± 2	0.2 ± 0.04	10 ± 2	20 ± 2	0.2 ± 0.04	10 ± 2	20 ± 2	0.2 ± 0.04	10 ± 2	20 ± 2
	4.87%	0.17%	−6.26%	3.47%	2.09%	1.68%	4.55%	3.48%	1.68%	[−9.65%; 19.39%]	[−12.24%; 12.59%]	[−10.18%; −2.34%]

**Table 2:** Nicotine consistency test in aerosols expressed as percentage deviation from the average value.

E-Liquid 1	E-Liquid 2	E-Liquid 3
−5.86	1.21	−0.660
−8.11 *	−3.43	−3.59
4.40	3.83	3.93
3.88	0.645	−0.756
5.70	−2.25	1.08

* outlier.

## Data Availability

The data are contained within this article.
